# A Community Outbreak of Legionnaires’ Disease with Two Strains of *L. pneumophila* Serogroup 1 Linked to an Aquatic Therapy Centre

**DOI:** 10.3390/ijerph19031119

**Published:** 2022-01-20

**Authors:** Cyril Rousseau, Christophe Ginevra, Leslie Simac, Noel Fiard, Karine Vilhes, Anne-Gaëlle Ranc, Sophie Jarraud, Hervé Gornes, Damien Mouly, Christine Campese

**Affiliations:** 1 The French Public Health Agency (Santé Publique France), Region Division—Occitanie, 31050 Toulouse, France; cyril.rousseau@santepubliquefrance.fr (C.R.); leslie.simac@santepubliquefrance.fr (L.S.); 2 The National Reference Center of *Legionella*, 69317 Lyon, France; christophe.ginevra@chu-lyon.fr (C.G.); anne-gaelle.ranc@chu-lyon.fr (A.-G.R.); sophie.jarraud@univ-lyon1.fr (S.J.); 3 CIRI, Centre International de Recherche en Infectiologie, Inserm, U1111, Université Claude Bernard Lyon 1, CNRS, UMR5308, Ecole Normale Supérieure de Lyon, 69007 Lyon, France; 4 The Regional Health Agency of Occitanie, 34000 Montpellier, France; noel.fiard@ars.sante.fr (N.F.); karine.vilhes@ars.sante.fr (K.V.); herve.gornes@ars.sante.fr (H.G.); 5 The French Public Health Agency (Santé Publique France), Infectious Disease Direction, 94415 Saint-Maurice, France; christine.campese@santepubliquefrance.fr

**Keywords:** *Legionella pneumophila*, outbreak, aquatic therapy centre, sequence typing

## Abstract

An outbreak of Legionnaires’ disease affected 18 people in Montpellier, a town of the south of France, between December 2016 and July 2017. All cases were diagnosed by a positive urinary antigen test. No deaths were reported. Epidemiological, environmental and genomic investigations (nested Sequence-Based Typing (nSBT) and whole genome sequencing) were undertaken. For the cases for which we had information, four had a new isolate (ST2471), one had a different new isolate (ST2470), one had a genomic pattern compatible with the ST2471 identified by nSBT (*fla*A = 3), and one had a genomic pattern not compatible with two previous identified STs (*pil*E = 6). The analysis conducted on the pool of an aquatic therapy center revealed seven isolates of *Legionella pneumophila*. Whole genome analysis confirmed the link between the environmental and clinical isolates for both ST2470 and ST2471. As the outbreak occurred slowly, with several weeks between new cases, it was not possible to immediately identify a common source. The sixth case was the first to report having aquatic therapy care. Of the 18 cases, eight had attended the aquatic therapy center and the other 10 were inhabitants who lived, worked or walked close to the center. The main cause for this outbreak was the lack of facility maintenance. This investigation highlights the risk to public health of aquatic therapy centers for users and nearby populations, and emphasizes the need for risk reduction measures with specific guidelines to improve health and safety in aquatic facilities.

## 1. Introduction

Legionnaires’ disease (LD) is a form of pneumonia caused by *Legionella*, a bacterium mostly present in man-made water systems. It is usually acquired by inhalation of contaminated aerosols [1,2]. For most of LD cases, the source of contamination cannot be definitively identified [3]. Control of LD outbreaks relies on descriptive epidemiological data, combined with microbiological information to identify the source and implement control measures.

In France, LD is mandatorily notifiable [4]; 1 218 LD cases were notified in 2016, with an annual notification rate of 1.8/100,000 inhabitants. Twenty-two percent of notified cases in 2016 had clinical isolates typed by the National Reference Centre for *Legionella* (NRC-L). Each confirmed case must be immediately interviewed by the local health authority in order to identify exposure risks and the likelihood of an outbreak, so that control and prevention measures can be implemented if necessary. The sources and numbers of cases in LD outbreaks are variable [2,3]. Previous outbreaks have been linked globally to a variety of aerosol-producing devices, including cooling towers, domestic water systems, water network systems in retirement/nursing homes, hospitals and tourist accommodations, as well as mist makers, decorative fountains, nebulizers and dental unit water line systems [5,6,7].

Following several large outbreaks of LD between 1998 and 2004 in France [8], new regulations were implemented for the various contexts listed above and spas [9].

Between January and July 2017, an outbreak of LD occurred in the city of Montpellier, in the south of France. Some of the cases lived in the same district of the city, while others worked or walked close to this district. This paper describes the related epidemiological, environmental and microbiological investigations conducted, as well as control measures put in place to control the outbreak. A particular attention has been made to the genomic investigations and of the comparison between the human and the environmental strains of *Legionella* in the aim of identified the causal source of contamination. The purpose of this study is to point out deficiencies and key preventive actions in a public health perspective.

## 2. Methods

### 2.1. Epidemiological and Microbial Investigations

Outbreak of LD is defined by the occurrence of at least 2 or more cases in a time and space area susceptible to involve a same source of exposure. A confirmed outbreak case was defined as a person with pneumonia and laboratory evidence of *Legionella* infection as per the national case definition [5], with illness onset between 15 December 2016 and 31 July 2017, who was living, visiting or working in the concerned district of Montpellier in the 14 days before illness onset. The district is located in the center of the Montpellier city included almost 9000 inhabitants living in a small area of 0.5 km².

The local health authority investigation team conducted interviews of all 18 confirmed cases or their relatives, using a standardized questionnaire which collected data on places in the city which they had visited in the previous 14 days. Active case finding of LD cases was also immediately implemented in hospitals and laboratories in Montpellier. Immediate notification of LD cases was requested by Santé Publique France (the French HPublic Health Agency). In addition, general practitioners in the affected district were asked to collect lower respiratory tract specimens and send them directly to the NRC-L. A descriptive analysis was performed to guide environmental investigations.

### 2.2. Environmental Investigation

On the basis of available data in the literature on the potential sources of exposure to Legionnaire’s disease, we aimed to identify a common source for the outbreak in Montpellier using in-depth environmental investigations.

All cases were invited to provide samples from domestic water systems for analysis, but only two did so. Databases from cooling tower systems (CTS), industrial plants, the services industry, and public facilities were all studied to identify potential local sources. These data were cross-checked using aerial views and local investigations. The environmental team of the local health authority then investigated the potential environmental sources of contamination and collected water samples for laboratory analysis. Among these sources, seven were CTS located in a four-kilometer radius of the district where all cases had been physically present. Three of these CTS were operational during the study period. An unannounced check was also requested by the local team.

In the past, street-cleaning trucks have been identified as potential sources of *Legionella* [10]. As these trucks cleaned the streets three days a week in the district because there was a local market, the investigation team had samples taken from ten cleaning trucks. Samples were also taken for an ornamental fountain close to the district that had recently been refilled, as well as from a car parking garage where stagnant water was found near a ventilation shaft that could have caused water spray.

Finally, because one case had received treatment in an aquatic therapy centre (ATC) with a spa pool in the district, samples were taken from this facility. This centre was located in a small building with other offices, and had an individual warm water distribution system to fill the pool.

Overall, a total of 30 samples from 20 potential sources were analysed. Detection and enumeration of *Legionella* in environmental samples was performed according to the French standard NF T90-431:2017 (similar to ISO 11731-2) culture method. *Legionella* strains from clinical and environmental samples and water samples were analysed by the NRC-L.

### 2.3. Genomic Investigation

*L. pneumophila* (Lp) isolates were typed at the NRC for *Legionella* using whole genome sequencing (WGS) with Nextera XT DNA Library Prep Kit (Illumina^®^, San Diego, CA, USA), and 2 × 300 bp paired-end reads using a MiSeq system (Illumina^®^). Genomes were assembled with an in-house pipeline using the Trimmomatic, KmerGenie, and SPAdes software packages. Sequence Types (ST) were extracted from WGS data using the mompS_tool pipeline and visualized using Bionumerics software platform 7.1 (Applied Math, St Martens Latem, Belgium) [11]. Core genome multilocus sequence typing (cgMLST) was extracted from WGS data using chewBBACA software; it included 1973 genes for this particular dataset [12].

Nested-PCR based Sequence-Based Typing (SBT) was performed on culture negative respiratory samples as described elsewhere [13].

## 3. Results

### 3.1. Epidemiological Results

**Figure 1:**
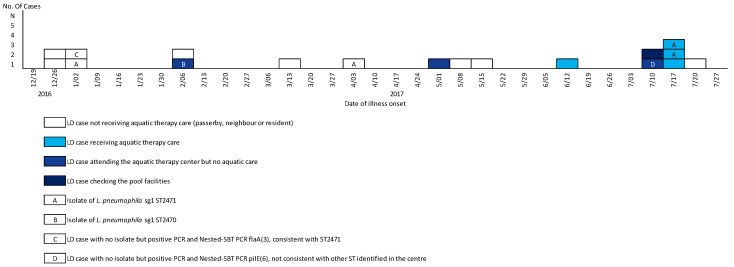
Outbreak of Legionnaires’ disease in a district in the city of Montpellier, December 2016 to July 2017 (*n* = 18). Potential exposures and dates of illness were obtained from patient interviews and microbiological analyses performed by NRC-L.

Eighteen patients fulfilled the case definition. Illness onset dates ranged from December 2016 to July 2017 (Figure 1). Ten were men (male/female ratio 1.25) and median age was 63 years (range; 41–93 years). Among them, 7 were living in the affected district and 11 outside of the district (in other district of the Montpellier City) but visited it and/or had an Aquatic therapy care during the incubation period (Table 1). Ten were men (male/female ratio 1.25) and median age was 63 years (range; 41–93 years). All 18 were living in the affected district or visited it during the incubation period. The attack rate was 140 cases/100,000 residents in the district. This long-term outbreak where several weeks passed before a new case appeared suggested an intermittent common source.

Between December 2016 and April 2017, only one of the eight confirmed cases to that point (case 5, Table 1) had visited the ATC. Between May 2017 and July 2017, seven of the remaining ten cases had visited the same ATC (Figure 1).

Fifteen of the 18 cases were hospitalized, with no fatalities. Associated risk factors were smoking (7/18 cases) and diabetes mellitus (4/18 cases). No underlying condition was identified for five cases. LD diagnosis for all 18 cases was performed by detecting the *Legionella* urinary antigen.

There were various potential sources for exposure to water droplets. This prevented immediate identification of a common source until the sixth case (symptom onset in February 2017) reported receiving aquatic therapy care. This case was followed by case nine (symptom onset in May 2017). Overall, seven cases had visited a physiotherapist studio (shared by several physiotherapists) in the same building as the ATC. Four of these received aquatic therapy by the same physiotherapist in the ATC during the incubation period. One of the 18 cases was a technical expert mandated for the ATC inspection. He became ill only a few days after his inspection. The analysis of the movements of the ten cases who did not attend the ATC showed that six of them went down the street where it was located at least once a week. One of these lived in an apartment directly above the ATC. The four other cases frequented the district at least once during the incubation period, although no more precise information was available.

### 3.2. Environmental and Microbial Investigation Results

#### 3.2.1. Potential Sources Investigated

Fifteen possible sources were identified from the databases examined and from local investigations. *L. pneumophila* was not isolated in any of the three CTS, in the ornamental fountain, in the 10 water tanks of the street-cleaning trucks or in the stagnant water of the car park. The two domestic water investigations also returned negative results.

#### 3.2.2. Aquatic Therapy Centre

The pool in the ATC had not been declared to sanitary authorities as required by the French Public health Code. In February 2017, the local health authority had samples taken in the ATC’s pool and water distribution system. In early May 2017, several system nonconformities were identified during a visit to the ATC by the local health authority investigation team: leaks under the pool, insufficient heating of water to a high enough temperature, no system logbook, and a lack of regular maintenance. However, *L. pneumophila* was not isolated in pool samples (*n* = 2) or in the water distribution system samples (*n* = 6) in February or in May of 2017 (Table 1). The shower in the physiotherapists’ studio located in the building was also sampled (May 2017), with Lp8 being isolated.

An air-to-air heat pump was located in the pool’s technical room, and both its air extraction duct and the pool’s moist air extraction duct vented onto the terrace of the apartment of one of the cases (case 4, Table 1). An air conditioning and heating technical expert was requested by the local health authority investigation team to carry out an inspection in July 2017. Many critical system issues were highlighted regarding the pool: no chlorine, leaks, hot water production lower than 50 °C, and controlled mechanical ventilation out of order.

With regard to the air-to-air heat pump, no exchange between air and water was possible, so it was not considered a high risk installation for *L. pneumophila*. The only areas sampled were the system’s condensation trays of the heat pump where stagnant water can appear under certain operating conditions (e.g., change of outside temperature). Samples tested negative (July 2017).

Fourteen supplementary samples from the ATC were analyzed in July 2017. Thirteen were non-quantifiable for *L. pneumophila* because of interfering flora. Thirteen *L. pneumophila* (12 Lp1 and 1 Lp5) were identified by NRC-L in samples from the pool and in a tray for pool chemical buffer (Table 2).

### 3.3. Genotyping Results

#### 3.3.1. Genotyping of Clinical Samples

Cultures of respiratory specimens were performed by the NRC-L for 12 of the 18 cases (67%). A positive culture was obtained for five of them (18%). Specifically, a new strain (ST2470) was isolated from a patient who had received physiotherapy care in the ATC, while a different new strain (ST2471) was isolated for two patients who had received aquatic therapy care, another who lived in the district, and finally one case who frequented the district but did not live there (Table 1).

*Legionella* PCR performed on the seven negative-culture respiratory samples were negative for three of them, and positive for the other four (cases 4, 13, XX, and YY). A nested sequence-based typing (nSBT) assay was performed for the latter. For case 4, 1/7 genes sequenced (allele *fla*A = 3) was consistent with the ST2471 strain. This case lived above the ATC with ducts from the ATC venting onto his terrace. For case 13, 1/7 gene was sequenced (allele *pil*E = 6) consistent with none of the other ST identified in the study. The case had received aquatic therapy care in the ATC. For the other two cases who received aquatic therapy care, no amplification for the seven genes was obtained.

#### 3.3.2. Genotyping of Environmental Samples

With regard to the 11 samples of *L. pneumophila* serogroup 1 (Lp1) identified from the pool and the tray for the chemical buffer, SBT and WGS identified ST2470 in one sample, ST2471 in seven samples (same as in four patients), and ST23 in three samples (Table 2). The Lp5 isolated from the chemical buffer sample was ST1324, while the Lp8 isolated from the physiotherapists’ studio shower sample in May 2017 was ST378. None of these ST (ST2470, ST2471, ST23, ST1324, ST378) were compatible with the *pil*E = 6 allele found from the one culture-negative patient (case 13).

**Figure 2:**
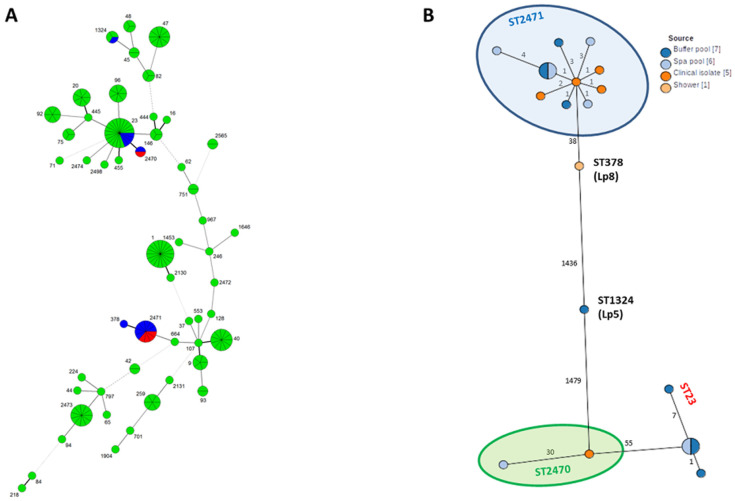
(**A**) Minimum spanning tree of 170 *Legionella pneumophila* Sequence Type isolated in 2017 based SBT allelic profiles (7 genes). Blue charts represent environmental isolates from the aquatic centre; red charts represent the isolates from the patients; green charts represent the other French isolates typed in 2017. Numbers beside each circle represent the sequence type (ST). (**B**) Minimum spanning tree of 19 *Legionella pneumophila* isolates from the epidemiological investigations based on cgMLST allelic profiles (1973 genes) extracted from whole genome assemblies using chewBBACA software.

SBT analysis comparing all known French ST during 2017 (170 isolates: 123 clinical and 47 environmental) identified two distinct clonal complexes each comprising two ST (Figure 2A). The clonal complex 1 comprised four clinical and three environmental Lp1 isolates belonging to ST2471 that were close to one Lp8 environmental isolate, ST378 (single locus variant of ST2471). Clonal complex 2 included one clinical and one environmental Lp1 isolate belonging to ST2470, which is genetically close to four Lp1 environmental isolates belonging to ST23 (single locus variant of ST2470). cgMLST analysis of the 19 isolates of the investigation confirmed the links between the clinical and environmental isolates of each clonal complex (Figure 2B). The ST of *L. pneumophila* serogroup 5 (ST1342) isolated from the ATC samples was not related to either of the two clonal complexes.

## 4. Discussion

This community outbreak of Legionnaires’ disease in a district in the city of Montpellier between December 2016 and July 2017 comprised 18 cases. The preliminary investigation found one common exposure factor, which was that all people affected lived, worked or frequented the district. Several potential sources were initially identified, but environmental microbiology highlighted no involvement. Investigations were refocused after two cases declared (at separate moments) they had received aquatic therapy care in an aquatic therapy center in the district. While two initial environmental samples taken from the pool tested negative in February and May 2017, related *L. pneumophila* were isolated from samples taken in the center in July 2017. This installation was then closed to the public by the ARS in July 2017 and no further cases were subsequently observed.

Three different *L. pneumophila* serogroups including five different ST were identified in the isolates from the ATC. Of these ST, two were also identified in clinical isolates. Both belonged to two new unrelated ST: ST2470 and 2471, respectively. WGS helped identify two distinct clonal complexes from clinical and environmental isolates, and confirmed the link between sample types for both. It is likely that both ST, as opposed to just one, contributed to the outbreak, as this was previously seen in a spa house in Japan which was colonized by several *L. pneumophila* strains [14]. This investigation highlights the need to encourage physicians to take respiratory specimens in order that the NRC can make in-depth molecular analyses.

The fact that this genotype was isolated in patients who received aquatic therapy care makes a strong epidemiological, microbiological and environmental argument that the pool in the center was the most probable source of the outbreak. Furthermore, the fact that two community cases (notified in December 2016 and April 2017)—who only walked close to the center but never entered it—were carrying the new strain with the same genotype as the strain isolated in the pool, is also a strong argument that the aquatic therapy center was responsible for the larger community outbreak.

For one patient, the partial genotyping result (*pil*E = 6) did not match any of the other genotypes identified from the center. One hypothesis for this is that the source of the infection may have been different for this patient. Another is that the overall genomic diversity of the *L. pneumophila* population present in the center was not fully identified by the analyses. Indeed, by typing only 13 isolates, we were able to identify five different STs belonging to three serogroups which were dispatched in three different genetic clades. Having a more complete picture of the genomic diversity of the center’s *L. pneumophila* population by genotyping more isolates would have allowed us to identify more ST, perhaps including ST compatible with the *pil*E allele 6 from this patient.

Many irregularities were identified in the ATC, including no chlorine in the pool, leaks, no maintenance logbook, and the fact that an air extraction duct of a heat pump was placed in the pool’s technical room and which vented next to the pool’s moist air extraction duct. However, the roles of these ducts remained unclear. Indeed, in a long-lasting outbreak in a hostel with a spa, pieces of air-conditioning filters tested positive for molecular biology, suggesting that the hostel’s air-conditioning system was probably implicated [15]. The expert assessment of the centre in our investigation suggested that it was possible that water droplets extracted outside through the pool’s air extraction duct could then be propelled further outside because the heat pump extraction duct was too close to it. Accordingly, we identified two possible exposure scenarios: (i) direct inhalation of droplets when attending the aquatic therapy care or when staying near the pool, and (ii) aerosol production outside the centre through the moist air extraction duct located on the roof, despite it not being located directly on the street.

Several LD outbreaks linked to spa pools or whirlpools have been described since the 1990s. The most sources of contamination in outbreaks in France were cooling towers. Aquatic therapy centre has never been identify as a source of contamination in outbreaks in France. The aeration and agitation of warm water, and the possibility of a biofilm developing in such installations is high: accordingly regular maintenance and disinfection procedures are essential. The detection of environmental *L. pneumophila* strains may be difficult in this type of device because whirlpool spas are routinely drained and disinfected or because *L. pneumophila* was only likely in certain components of the devices (filters, etc.). In our case, pool samples tested negative twice for *L. pneumophila* in February and in May 2017, and many of our cases did not attend the ATC during the first period (January to April 2017). Had biofilm swabs been used for sampling in certain areas of the centre, it might have helped detect *L. pneumophila* sooner [15], and consequently shortened the duration of this outbreak (i.e., through interventions like closing the centre, etc.).

The literature highlights that persons need only be physically close to a whirlpool spa—even simply walking past one—to become infected, because of the potential for a dispersion of the droplets in the air [16,17]. This situation of aerosolization may generate large outbreaks [18]. In our case, despite a high number of people potentially exposed in this district over six months, the outbreak was of modest size, with periods of several weeks between new cases. This may be explained by possible discontinuous and low contamination of the pool, or by discontinuous production of droplets outside the premises, for example, due to meteorological conditions.

## 5. Conclusions

This unusually long-lasting community outbreak of Legionnaires’ disease presented special challenges. More specifically, difficulties in identifying the source and strains required actors to refocus investigations, which led to an extended response time before controlling the outbreak. In France, spa pool installations must be systematically reported to health authorities as they may be involved in community LD outbreaks under certain circumstances. While the causes of LD clusters and outbreaks are in general rarely identified, perhaps partly because CTS are increasingly being replaced by adiabatic systems, spa pool exposure should be included in legionellosis community cluster and outbreak investigations, even when evidence is not initially available. This is especially true given that aquatic therapy centers are frequently visited by elderly people with specific medical conditions who constitute a particularly vulnerable population.

## Figures and Tables

**Table 1:** Epidemiological and microbiological characteristics of patients with outbreak-related L. pneumophilia serogroup 1 from a district in the city of Montpellier, December 2016 to July 2017 (*n* = 18).

Date of Illness	Status	Aquatic Therapy Care	*L. pneumophila* Urinary Antigen Testing	ST Subtyping	Pulsed-Field Gel ElectroPhoresis (PFGE)	Monoclonal Antibodies to *Legionella* Proteins (Mabs)	Nested-*Polymerase Chain Reaction* (PCR)
2016-51	passersby	no	(+)	-	-	-	-
2016-51	passersby	no	(+)	-	-	-	-
2016-52	passersby	no	(+)	2471	Sporadic **	FRA/ALL	-
2016-52	neighbour	no	(+)	consistent 2471	-	-	*fla*A(3)
2017-05	aquatic center patient	yes	(+)	2470	Louisa	FRA/ALL	-
2017-05	resident	no	(+)	-	-	-	-
2017-10	passerby	no	(+)	-	-	-	-
2017-13	working in the area	no	(+)	2471	Sporadic **	FRA/ALL	-
2017-18	passerby		(+)	-	-	-	-
2017-17	aquatic center patient	yes	(+)	-	-	-	negative
2017-19	passerby	no	(+)	-	-	-	ND
2017-23	aquatic center patient	no aquatic care *	(+)	-	-	-	ND
2017-27	aquatic center patient	yes	(+)	-	-	-	*pil*E(6)
2017-27	aquatic center visitor	facilities inspection	(+)	-	-	-	negative
2017-28	aquatic center patient		(+)	-	-	-	ND
2017-28	aquatic center patient	no aquatic care *	(+)	2471	Sporadic **	FRA/ALL	-
2017-29	resident	no	(+)	-	-	-	-
2017-28	aquatic center patient	no aquatic care *	(+)	2471	Sporadic **	FRA/ALL	-

* only physiotherapy; ** sporadic: already identified by the NRC; (+) positive; ND: no data.

**Table 2:** Environmental microbiology results, outbreak of Legionnaires’ disease, district in the city of Montpellier, December 2016 to July 2017.

	Local Laboratory Results		Typing of Strains by NCR *Legionella*			
Location	Quantification of *Legionella* in Environmental Samples	Date		*L. pneumophila Serogroup*	ST Subtyping	Date
Car park	<10 cfu/L	April 2017	Aquatic therapy center			
Ornamental fountain	*L. pneumophila* undetectable	April 2017	spa pool	1	23	July 2017
Street-cleaning trucks (*n* = 10)	*L. pneumophila* undetectable	May 2017	spa pool	1	2470	July 2017
CTS (*n* = 3)	<10 cfu/L	May 2017	spa pool	1	2471	July 2017
			spa pool	1	2471	July 2017
Aquatic therapy center			spa pool	1	2471	July 2017
Hot water production	*L. pneumophila* undetectable	February, May 2017	spa pool	1	2471	July 2017
Hot water point-of-use	*L. pneumophila* undetectable	February, May 2017	Buffer pool	1	23	July 2017
Cold water	*L. pneumophila* undetectable	May 2017	Buffer pool	1	23	July 2017
Condensation products *	*L. pneumophila* undetectable	July 2017	Buffer pool	1	23	July 2017
Spa pool	<10 cfu/L	February, May 2017	Buffer pool	1	1324	July 2017
Spa pool	non-quantifiable (interferent flora)	July 2017	Buffer pool	1	2471	July 2017
Buffer pool	nd	February, May 2017	Buffer pool	1	2471	July 2017
Buffer pool	non-quantifiable (interferent flora)	July 2017	Buffer pool	1	2471	July 2017
Shower	*L. pneumophila*: 160 cfu/L	May 2017	Shower	8	378	May 2017

CTS: cooling tower system; cfu: colony forming units; nd: not done; * terminal units air-to-air heat pump.

## Data Availability

Data are available at Santé publique France.
